# Fusion zone microstructure image dataset of the flux-cored and shielded metal arc welding processes

**DOI:** 10.1016/j.dib.2020.106353

**Published:** 2020-09-30

**Authors:** Moisés Luiz Lagares Jr, Gulliver Catão Silva, Lecino Caldeira

**Affiliations:** a Faculty of Engineering, Federal University of Juiz de Fora, Juiz de Fora, Brazil; b Federal Institute of Southeast of Minas Gerais State – IF Sudeste MG, Juiz de Fora, Brazil

**Keywords:** Welding process, Welding metallurgy, Steel microstructure, Computational intelligence, Pattern recognition, Machine learning

## Abstract

This paper presents high quality (2048 × 1532 pixels) Light Microscope steel images sampled from the welding fusion zone. The microstructure images were acquired from the Design of Experiments (2^2^ full factorial design) planned to compare two different arc welding processes at two different arc welding energies [1]. The 400 raw images appear as they were captured by the microscope and they are categorized into four groups: that acquired from the Flux Cored Arc Welding process and that acquired from the Shielded Metal Arc Welding process; both of them run for high and low levels of arc energy. For the Flux Cored Arc Welding process, ASME SFA 5.20 E71T-5C(M) tubular wire was used, with a nominal diameter of 1.2 mm. For the Shielded Metal Arc Welding process, AWS E7018 coated electrodes were used, with nominal diameters of 3.25 mm (for the low energy level) and 5.00 mm (for the high energy level). The deposition of the beads was run on AISI 1010 steel plates in the flat position (bead-on-plate). Different proportions of primary grain boundary ferrite; polygonal ferrite; acicular ferrite; nonaligned side-plate ferrite and aligned side-plate ferrite can be observed in each image. This image dataset is ready to visual and automatic microstructure recognition and quantification. It can be a useful resource for computational intelligence research teams, e.g. [2], by offering images for handling as filtering, feature extraction, training, validation and testing in pattern recognition and machine learning techniques.

## Specifications Table

**Table:** 

Subject	Metals and Alloys
Specific subject area	Welding metallurgy and welding processes
Type of data	Image
How data were acquired	Light Microscope
Data format	Raw
Parameters for data collection	400 fusion zone microstructure images were collected from the Design of Experiments (D.O.E.) involving two different welding processes at two different energy levels. The conditions considered for each welding process and the D.O.E. are described in Section 2 as well as the energy levels.
Description of data collection	The images were captured by the digital camera of the Light Microscope Olympus GX51. The embedded imaging analysis software systems were used to labeling each image with the dimension reference in microns (Scale Bar). All images were obtained in Bright Field Mode.
Data source location	Metallography Laboratory of the Federal Institute of Southeast of Minas Gerais State – IF Sudeste MG Juiz de Fora/Minas Gerais State Brazil
Data accessibility	Mendeley Data http://dx.doi.org/10.17632/zgchnmjkj7.1
Related research article	G.C. Silva, J.A. de Castro, R.M.M. Filho, L. Caldeira, M.L. Lagares Jr, Comparing two different arc welding processes through the welding energy: a selection analysis based on quality and energy consumption, Journal of the Brazilian Society of Mechanical Sciences and Engineering, 41 (2019). https://doi.org/10.1007/s40430–019–1804-x

## Value of the Data

• More than 400 high quality steel microstructure images from welding fusion zone are available for visual reference and for training, validation and test dataset in pattern recognition and machine learning techniques
• This image dataset is a useful resource for computational intelligence, metallurgical, mechanical and welding researchers and engineers
• This light microscope image dataset is ready to visual and automatic microstructure recognition and quantification; filtering and feature extraction; becoming an specific image dataset resource to be used in the efforts employed in the computational intelligence algorithms advances and automatic microstructures recognition

## 1 Data Description

This data image is categorized into four groups: that acquired from the Flux-Cored Arc Welding (FCAW) process and that acquired from Shielded Metal Arc Welding (SMAW) process; both of them run for high and low levels of arc energy. The applied Design of Experiments (D.O.E.), materials (filler and base metals) and methods are presented in the Section 2. Each group of data image presents one replication, making up a dataset of 400 images. They were acquired by Light Microscope (LM) and revealed the weld bead microstructure obtained for each welding process at different arc energy levels. Those groups present high quality LM images (2048 × 1532 pixels) of the microstructure found in the Fusion Zone (FZ) of the weld bead. Different proportions of primary grain boundary ferrite (PF(G)); polygonal ferrite (PF(I)); acicular ferrite (AF); nonaligned side-plate ferrite (FS(NA)) and aligned side-plate ferrite FS(A) can be observed in each image. The data description of each image group is presented as follows:

### 1.1 Group 1: fcaw (–)

**Fig 1:**
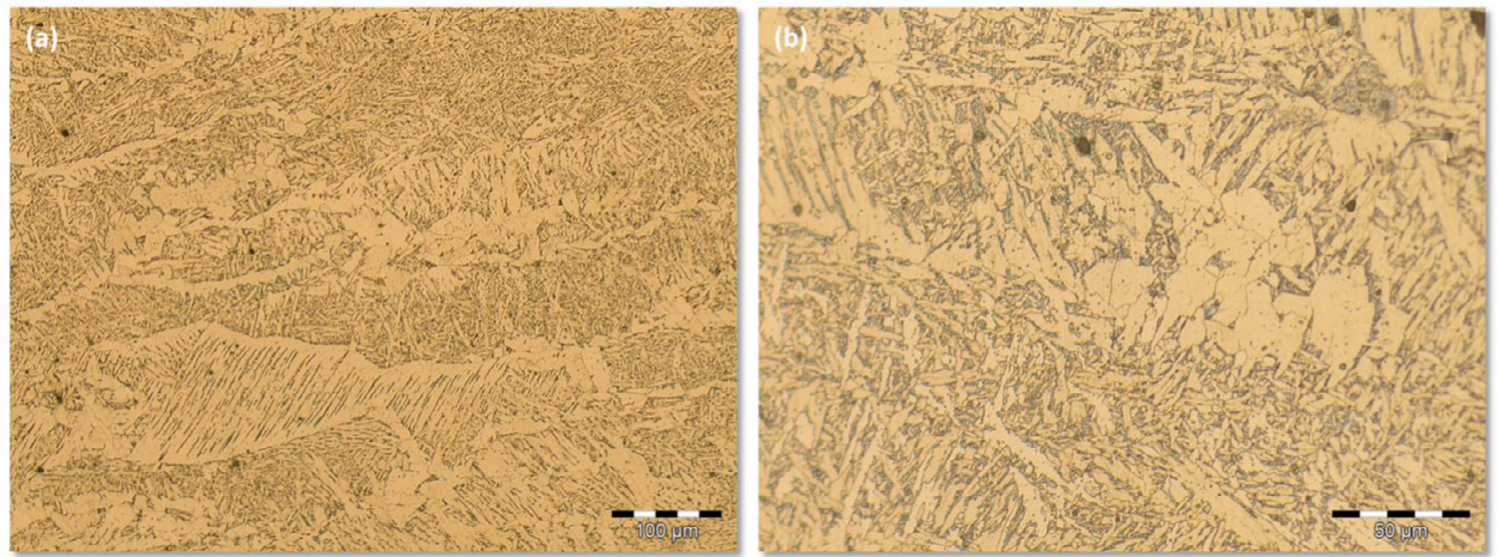
Fusion Zone microstructure for the FCAW process at low energy level: (a) 200X LM magnification; (b) 500X LM magnification.

Group that encompasses the images obtained from the FCAW process at low energy level. Fig. 1(a) shows a sample applying LM 200X magnification. It can be downloaded from the supplementary data files FCAW (-) archive. This supplementary data files archive also contain 50 images, at the same conditions, sampled from different locations of the same FZ. All of the 50 images were captured upon 500X LM magnification. Fig. 1(b) shows a sample with the higher magnification of 500X.

Another 50 images were sampled from the FCAW process replication and can also be downloaded from the supplementary data files *FCAW (-) Replication* archive, totalizing 100 image samples available for downloading referring to the conditions of the Group 1.

**Fig 2:**
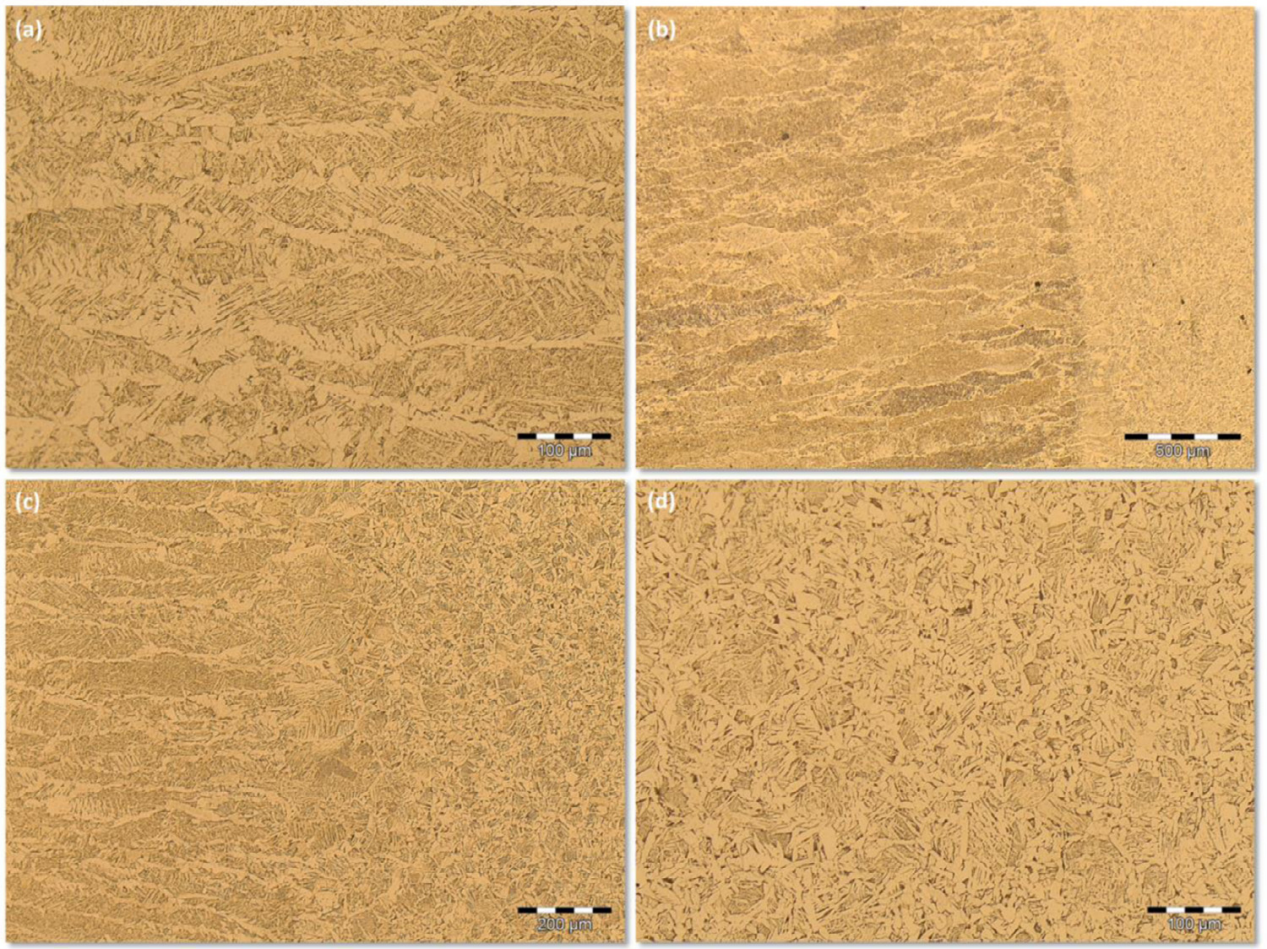
Microstructure for the FCAW process at low energy level: (a) FZ at 200X LM magnification; (b) the transition microstructure from FZ to HAZ (50X); (c) the transition microstructure from FZ to HAZ at higher magnification (100X) and the HAZ microstructure (200X).

Further images can be found in the supplementary data files. Fig. 2 exhibits a sequence of images showing an FZ microstructure available on the *FCAW (-) Replication* supplementary data files: (a) the FZ at 200X LM magnification; (b) the transition microstructure from FZ to the Heat Affected Zone (HAZ); (c) the transition microstructure from FZ to HAZ at higher magnification and (d) the HAZ microstructure.

**Fig 3:**
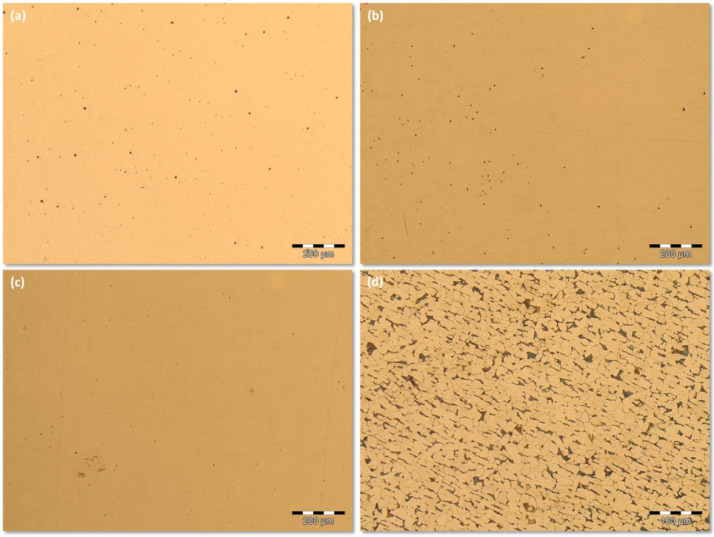
Inclusions distribution, before the etching, on the: (a) FZ; (b) transition microstructure from FZ to HAZ and (c) BM. (d) shows the microstructure of the etched BM.

Additionally, Fig. 3 exhibits an image sequence showing the inclusions, before the etching, on the FZ (a); on the transition microstructure from FZ to HAZ (b); on the Base Metal (BM) (c) and the etched BM (d).

### 1.2 Group 2: fcaw (+)

**Fig 4:**
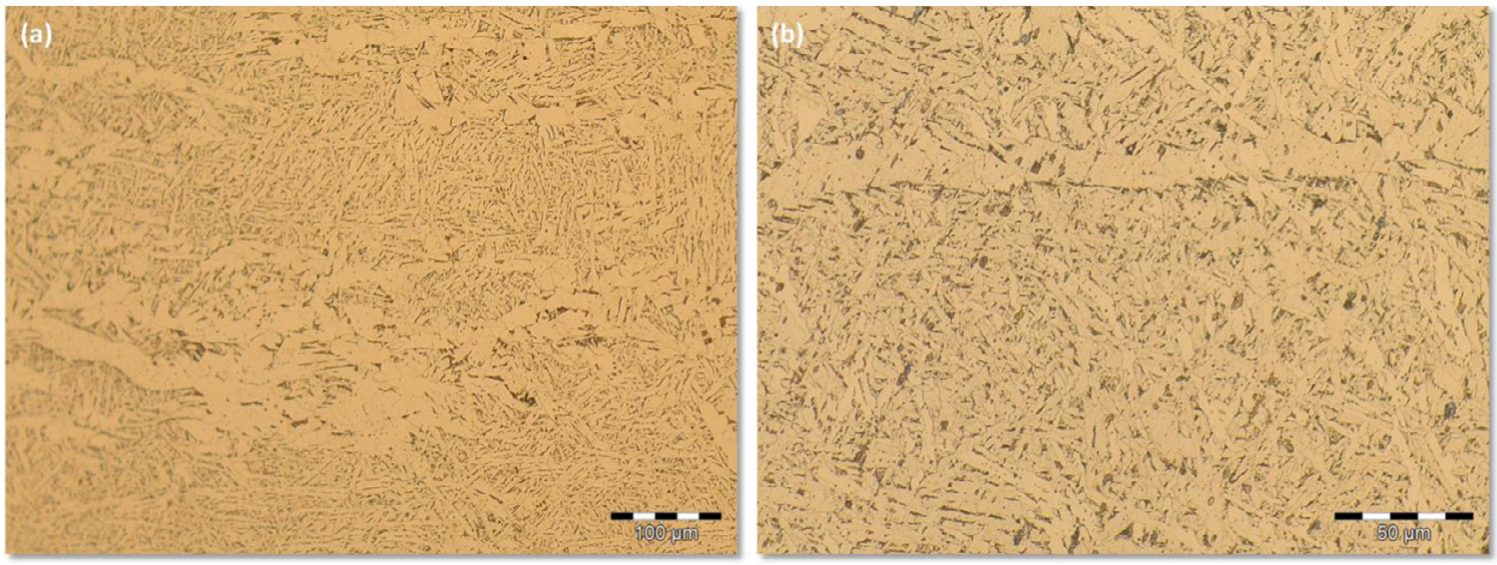
FZ microstructure for the FCAW process at high energy level: (a) 200X LM magnification; (b) 500X LM magnification.

Group 2 encompasses the images obtained from the FCAW process at high energy level. Fig. 4(a) shows one sample at 200X LM magnification. It can be downloaded from the supplementary data files *FCAW (+)* archive. This archive supplementary data files contain 50 images collected, at the same conditions, from different locations of the same FZ. All of the 50 images were captured upon 200X LM magnification. Fig. 4(b) is the sample obtained from the FCAW process replication at high energy level and can be downloaded from the supplementary data files *FCAW (+) Replication* archive, were another 50 images can be found but, here, upon 500X LM magnification.

### 1.3 Group 3: smaw (–)

**Fig 5:**
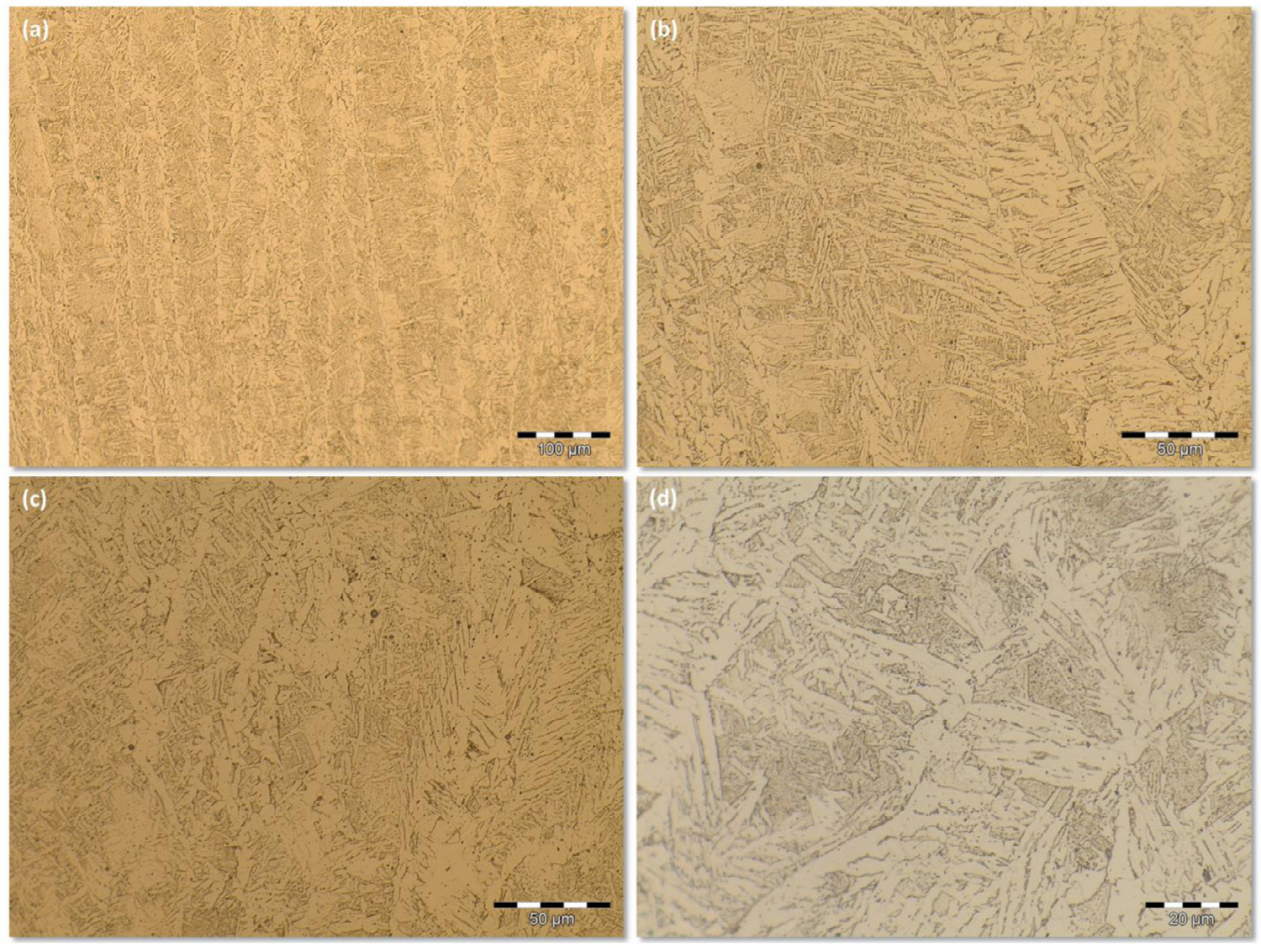
Fusion Zone microstructure for the SMAW process at low energy level and different LM magnifications: (a) 200X; (b) and (c) 500X, sampled from different location of the FZ; (d) 1000X.

**Fig 6:**
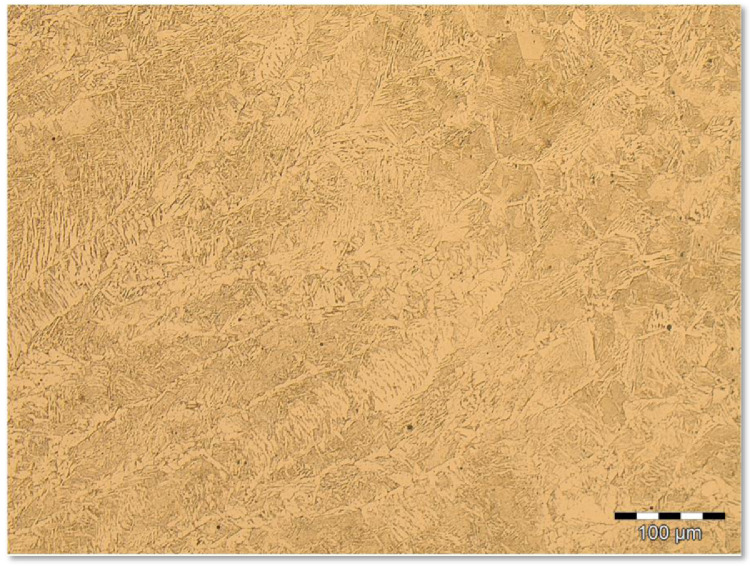
The transition microstructure, for the SMAW process at low energy level, from FZ to HAZ (200X).

Group that encompasses the images obtained from the SMAW process at low energy level. Fig. 5(a) shows a sample at the LM 200X magnification. Figs. 5(b) and (c) are microstructures at higher magnification (500x) taken from different location of the FZ. Fig. 5(d) presents the microstructure of the FZ at the LM 1000X magnification. They are available by downloading from the supplementary data files *SMAW (-)* archive and its respective *Replication* data archive. Both supplementary data files archives offer 100 images sampled, at the same welding process conditions, sampled from different locations of the same FZ. All of the 100 images were captured upon 500X LM magnification. Fig. 6 shows the transition zone from the FZ to HAZ at the 200X LM magnification.

### 1.4 Group 4: smaw (+)

**Fig 7:**
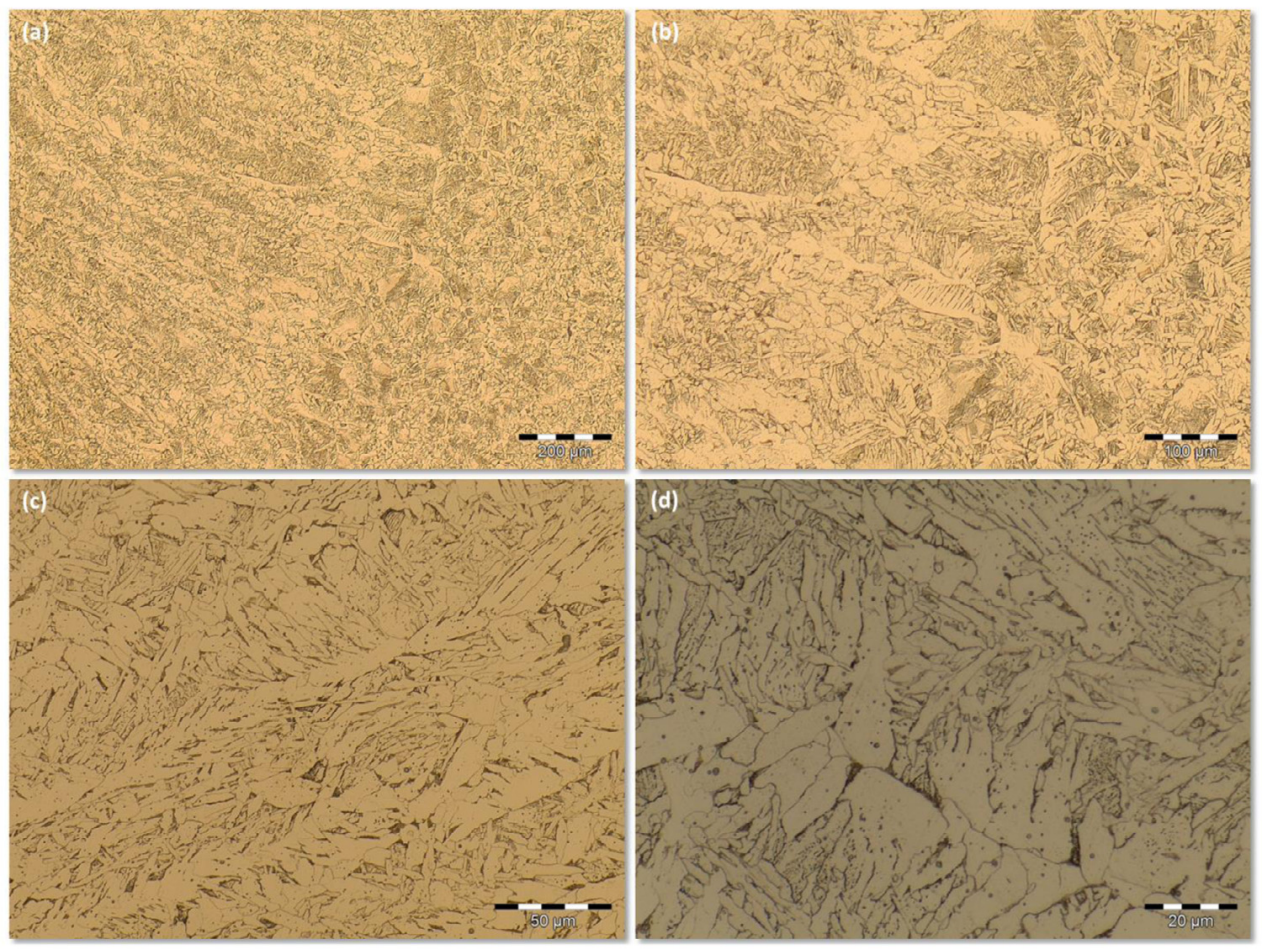
Microstructure for the SMAW process at high energy level and different LM magnifications: (a) the transition from FZ to HAZ at 100X; (b) the same location of Fig. 7(a) but at the higher magnification 200X; (c) the microstructure found on the FZ at 500X; (d) the microstructure of the FZ for the higher magnification 1000X.

Group 4 encompasses the images obtained from the SMAW process at high energy level. Fig. 7 shows a sequence of increasing LM magnification of images. Figs. 7(a) shows the transition from FZ to HAZ at 100X LM magnification. Fig. 7(b) presents the same location of Fig. 7(a) but at the higher magnification 200X. Fig. 7(c) is the microstructure found on the FZ for the 500X magnification while the Fig. 7(d) shows the microstructure of the FZ for the higher magnification of 1000X. They are available by downloading from the supplementary data files *SMAW (+)* archive and its respective *Replication* data archive. Both supplementary data files archives also offer 100 images, at the same welding process conditions, sampled from different locations of the FZ. All of the 100 images were captured upon 500X LM magnification.

## 2 Experimental Design, Materials and Methods

The image data was obtained from the D.O.E. presented in Table 1. That D.O.E. was planned in order to compare two different arc welding processes at two different arc welding energies (E), which was discussed by [1]. The compared welding processes were SMAW and FCAW. It is a 2^2^ full factorial design, with one replication. The factor 1 is the welding process and the factor 2 is the arc energy level. Table 1 also shows the wire feed speed (WFS) and the travel speed of the welding torch (S) applied in each trial. That design allowed producing four distinct image dataset that was described in Section 1. The respective mean welding current (I_mean_) and arc voltage (U_mean_) are also showed and the E is calculated by eq. (1):

(1) $$E=\frac{P_{inst}}{S}.10^{-3}\;\left[\frac{kJ}{mm}\right]$$

where *P_inst_* is the instantaneous power, in Watts, given by eq. (2) and *S* is the travel speed in millimeter per second.

(2) $$P_{inst}=\frac{1}{n}\sum_{n=1}^{n}\left(U_{i}.I_{i}\right)\;\left[W\right]$$

where *I_i_* and *U_i_* are the instantaneous current and voltage sampled values and *n* is the number of samples. The replications were done on different plates and the trial sequence was randomized.

**Table 1:** Design of experiments, welding parameters and the dataset label for each trial.

Dataset label	Trial	Factor 1	Factor 2	WFS (m/min)	I_mean_ (A)	U_mean_ (V)	S (cm/min)	E obtained (kJ/mm)
SMAW(–)	1	–	–	—	136.9 ± 13	23.7 ± 4.0	17.8	1.08 ± 0.18
FCAW(–)	2	+	–	7.0	172.4 ± 23	27.3 ± 1.2	28.0	1.01 ± 0.14
SMAW(+)	3	–	+	—	234.5 ± 6.7	26.3 ± 4.6	22.2	1.67 ± 0.28
FCAW(+)	4	+	+	10.0	247.9 ± 15	30.0 ± 0.8	28.0	1.65 ± 0.09

Replication	1	–	–	—	137.0 ± 15	23.4 ± 4.6	17.8	1.07 ± 0.18
	2	+	–	7.0	183.0 ± 18	27.3 ± 0.9	28.0	1.07 ± 0.11
	3	–	+	—	228.0 ± 5.5	26.4 ± 4.3	22.2	1.59 ± 0.25
	4	+	+	10.0	252.8 ± 2.2	30.4 ± 0.7	28.0	1.60 ± 0.10

		Factor 1 – Process:			(–) SMAW (+) FCAW.			
		Factor 2 – Arc Welding Energy:			(–) Low (+) High.			

The experimental procedure was run applying a multi-process commercial secondary chopper electronic power source. For the FCAW process, ASME SFA 5.20 E71T-5C(M) tubular wire was used, with a nominal diameter of 1.2 mm, torch inclination angle at 90°, Ar + 20% CO_2_ shielding gas at 11 L/min flow rate, contact tip-to-work distance at 19 mm, constant-voltage power source mode, reverse polarity (DC+). For the SMAW process, AWS E7018 coated electrodes were used, with nominal diameters of 3.25 mm (for the low E level) and 5.00 mm (for the high E level), constant-current power source mode, reverse polarity. The weld deposition was done on low-carbon AISI 1010 steel plates (250 mm x 50 mm x 6 mm), in the flat position (bead-on-plate). All the weldments were mechanized and performed with the aid of an automatic positioning and displacement system (automatic travel speed) of the welding torch and the electrode holder. In the SMAW welding, the technique of dragging the electrode over the base metal at a 45° angle was chosen [1].

The low (around 1.1 kJ/mm) and the high (around 1.7 kJ/mm) levels of *E* (Table 1) were obtained in pre-tests based on successive adjustments of the current, voltage and travel speed variables. Since the E7018 consumable welding current range is more restricted to a given electrode diameter, the choice was to use 3.25 mm (low energy level) and 5.00 mm (high energy level) coated electrodes to follow the current range used for the tubular wire. Instantaneous values of arc voltage, welding current, gas flow rate and wire feed speed were recorded during the weldments by an commercial data acquisition system at a sample frequency of 5 kHz and 10 bits resolution (0.2 V and 1.2 A resolution). Samples were taken from the welded plates for micrographic analyses, by light microscope, of the fusion zone cross section.

Metallographic samples preparation involved the sanding steps using the silicon carbide (SiC) sandpapers with 150, 220, 320, 400, 500, 600, 1200 and 13 μm grain sizes. Shortly after that, the samples were cleaned in an ultrasonic bath with distilled water by 15 min and subsequently polished in diamond past using the sequence 7, 3 and 1 µm along 15 min. Right after, samples were washed in distillated water and dried by a thermal blow.

Fresh 3% nital etchant was prepared using ethyl alcohol PA and nitric acid PA before each etching step. The etching time was 3 s [1]. The acquired images are a useful resource for computational intelligence research teams, e.g. [2], by offering images for handling as filtering, feature extraction, training, validation and testing in pattern recognition and machine learning techniques.

## Declaration of Competing Interest

The authors declare that they have no known competing financial interests or personal relationships which have, or could be perceived to have, influenced the work reported in this article.
